# Youth Exposure to Marijuana Advertising in Oregon’s Legal Retail Marijuana Market

**DOI:** 10.5888/pcd17.190206

**Published:** 2020-09-24

**Authors:** Steven C. Fiala, Julia A. Dilley, Erik M. Everson, Caislin L. Firth, Julie E. Maher

**Affiliations:** 1 Health Promotion and Chronic Disease Prevention Section, Oregon Public Health Division, Portland, Oregon; 2 Oregon Public Health Division and Multnomah County Health Department — Program Design and Evaluation Services, Portland, Oregon

## Abstract

**Introduction:**

Research from tobacco and alcohol markets suggests advertising exposure is associated with perceptions of lower risk and increased use among young people. Limiting marketing may be a regulatory approach to prevent potential negative effects of retail marijuana legalization on youth use. This study assessed marijuana advertising exposure reported by youths in Oregon after the start of retail marijuana sales in October 2015.

**Methods:**

Data from a 2017 school-based survey of Oregon 8th (N = 14,852) and 11th (N = 11,895) graders were used to characterize marijuana advertising exposure. Subgroup differences in reported exposure were assessed by using Pearson χ^2^ tests and multiple logistic regression.

**Results:**

About three-quarters of 8th (72.2%) and 11th graders (78.1%) in Oregon reported seeing marijuana advertising in the past month. Youths most frequently reported seeing advertising on storefronts and online, and odds of exposure were significantly higher for girls; lesbian, gay, or bisexual youths; current marijuana users; 8th graders living with an adult who uses marijuana; and youths in school districts with a closer average proximity to retail marijuana stores.

**Conclusion:**

Reporting exposure to marijuana advertising is common among youths in Oregon’s legal retail market. Oregon and other states working to prevent youth marijuana use may want to examine how well their rules are working to prevent youth exposure. Although some sources of youth advertising exposure may be difficult to regulate and enforce (eg, online), others may be within the purview of state authority (eg, billboards, storefronts) depending on state-specific interpretation of free speech protections.

**Summary**

**What is already known about this topic?**

Research from tobacco and alcohol markets suggests advertising exposure is associated with perceptions of lower risk and increased use among young people.

**What is added by this report?**

We assessed marijuana advertising exposure among youths in Oregon. About three-quarters of youths reported exposure to marijuana advertising. Exposure was higher among girls than boys; lesbian, gay, or bisexual youths than straight youths; and youths in school districts with a closer average proximity to retail marijuana stores.

**What are the implications for public health practice?**

Groups with higher exposure to advertising may benefit from targeted prevention efforts or countermessaging to delay initiation of marijuana use.

## Introduction

As of 2018, voters in 8 US states had legalized the production, processing, distribution, and sale of retail (ie, nonmedical) marijuana (ie, cannabis) for adults aged 21 years or older. One regulatory approach to prevent potential negative impacts of legalization on youths is limiting marijuana marketing, given that research from tobacco and alcohol markets suggests that advertising exposure is associated with lower perceptions of risk and substance use initiation among young people (1,2). Early evidence suggests that the same may be true for marijuana advertising: adolescents in California who were exposed to medical marijuana advertising had twice the odds of marijuana use and intention to use marijuana 1 year later compared with those who were never exposed (3).

Only one study to date has assessed population-based marijuana advertising exposure among US adolescents: Dai used Monitoring the Future data from 2014–15 and found that 58.7% of respondents reported some level of exposure in recent months (4). However, this study was conducted when only Colorado and Washington State had legal retail marijuana markets and the analysis did not account for the legal status of marijuana (eg, whether retail or medical sales were allowed). Studies of adults also suggest high levels of advertising exposure in the United States. In a 2015 national sample of young adult marijuana users aged 18 to 34 years, 66% of those living in states with legal retail markets reported exposure in the past month to marijuana advertising compared with 47% in states with legal medical markets and 46% in states with no legal markets (5). A study in Oregon found that after retail marijuana sales began in 2015, most adults in the state reported seeing marijuana ads, particularly in communities with stores selling retail marijuana compared with communities without stores (56.5% vs 32.5%, *P* < .001); exposure to marijuana advertising was even higher (63.2%) among young adults aged 18 to 20 years who were not of legal age to possess retail marijuana (6).

The objectives of our study were to estimate the prevalence of self-reported exposure to marijuana advertising among Oregon 8th and 11th graders after the opening of a legal retail marijuana market in October 2015, describe the source of marijuana advertising exposure (eg, online, storefront), and determine whether certain demographic characteristics and retail store quantity and proximity were associated with marijuana advertising exposure.

## Methods

### Data source

We used data from the 2017 Oregon Healthy Teens Survey (OHT) (7) for this study. The OHT is a school-based survey of Oregon 8th and 11th graders conducted in odd-numbered years and designed to measure the health and well-being of youths. OHT is anonymous and voluntary and is given via paper or online in school settings. All Oregon public secondary schools are included in the sampling frame. School districts are randomly sampled; in larger districts, schools are also randomly sampled from within those districts. OHT data are weighted to achieve a statewide representative sample based on the probability of students being selected. The 2017 OHT was administered in February through May 2017, which was more than 1 year after Oregon allowed retail marijuana sales through existing marijuana medical dispensaries (October 2015), and several months after licensed retail marijuana stores opened (October 2016). A total of 14,852 8th graders and 11,895 11th graders completed the survey, corresponding to an overall response rate of 63%; 84 of Oregon’s 187 eligible public school districts contributed data.

### Measures

**Figure 1:**
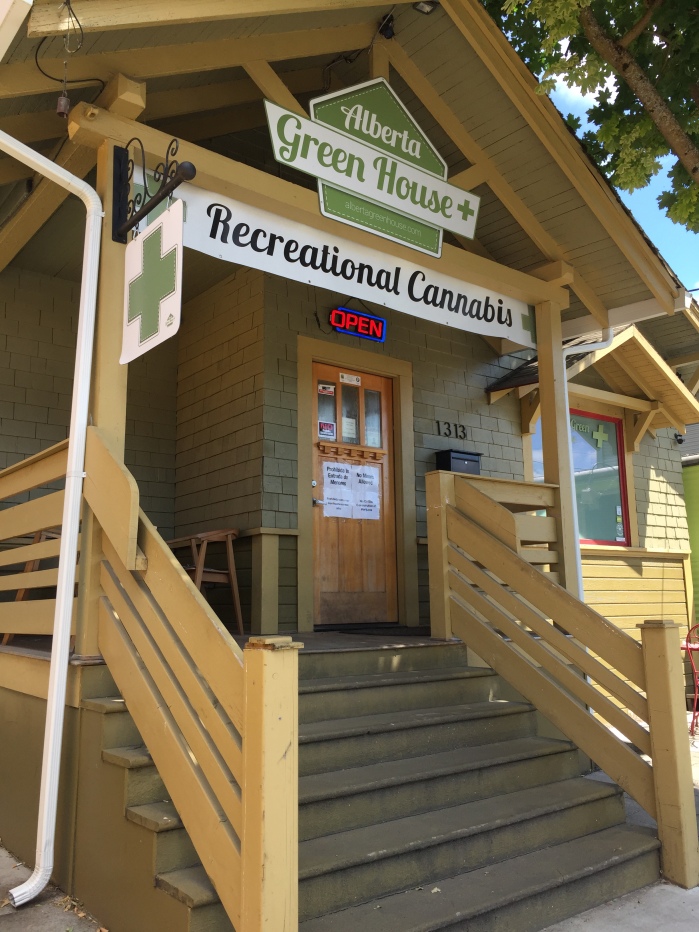
Retail marijuana storefront with green cross and sign advertising “Recreational Cannabis,” Oregon, 2017

**Figure 2:**
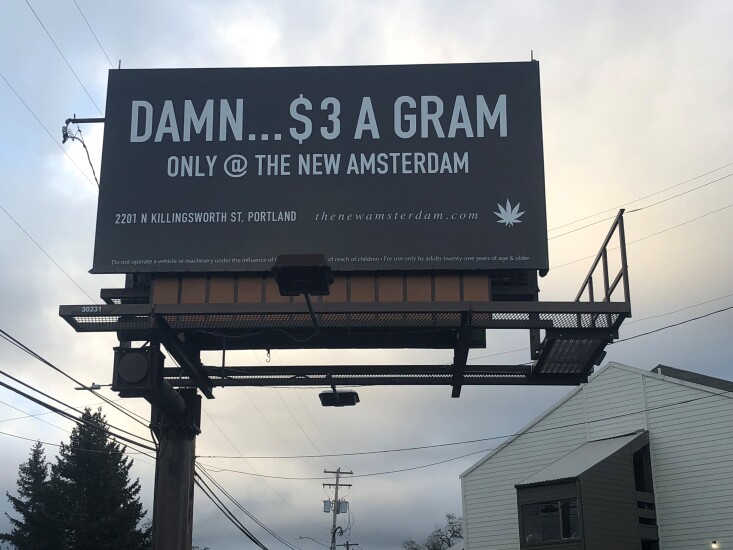
Billboard advertising retail marijuana, Oregon, 2017

Questions on exposure to marijuana advertising were added to the 2017 survey and were based on existing OHT questions related to tobacco advertising exposure. Respondents were asked “During the past 30 days, have you seen an advertisement for marijuana products or stores . . . 1) in a magazine or newspaper; 2) on a storefront; 3) online, on your cellphone, tablet, or computer through email, websites, or social media; 4) on a billboard; 5) on the sidewalk (like signs or people wearing or waving signs).” (A storefront advertising example is shown in Figure 1 and an example billboard is shown in Figure 2.) Respondents could select yes, no, or don’t know/not sure for each of the exposure types. Responses of don’t know/not sure were retained in the denominator for analysis of exposure to specific advertising types and the number of exposure sources. Students were classified as having any past-month exposure to advertising if they provided a yes/no answer to all 5 questions and responded yes to at least 1 of the 5 advertising types assessed.

Demographic variables were recoded for analysis. Respondent gender was categorized as female, male, or nonbinary/gender nonconforming. Sexual orientation was categorized as either straight or lesbian, gay, or bisexual (LGB). Race/ethnicity was categorized as non-Hispanic White, non-Hispanic American Indian or Alaska Native, non-Hispanic Asian, non-Hispanic Black or African American, non-Hispanic Native Hawaiian or other Pacific Islander, non-Hispanic multiple races, non-Hispanic other race, or Hispanic or Latino (any race). Community setting (urban, rural, or frontier residency) was assigned based on student self-reported zip code by using designations from the Oregon Office of Rural Health based on distance to population centers and population density. Zip code information was missing for 13.7% of 8th graders (n = 2,028) and 5.5% of 11th graders (n = 651), and they were consequently excluded from analysis of ad exposure by community setting.

We assessed current marijuana use with the question “During the past 30 days, on how many days did you use marijuana or hashish (weed, hash, pot)?” We considered respondents to be current users if they indicated at least 1 day of marijuana use in the past 30 days. We assessed exposure to marijuana use in the home with the question “Does any adult living in your house use marijuana?” Response options were yes and no.

The number of active marijuana retail licenses used in the proximity measure was obtained from the Oregon Liquor Control Commission website. We included active licenses as of March 2017 to align with timing of OHT data collection. We characterized exposure to retail marijuana stores in 2 ways. First, we described retail marijuana store exposure as the number of retail stores in the school district by using 3 cut points resulting in similar sample sizes: no stores, 1 to 3 stores, and 4 or more stores. Second, we defined and categorized proximity to retail marijuana stores by using methods previously developed by our team to describe marijuana retailer proximity (8). We assigned a proximity value to each OHT participant based on average distance to the nearest marijuana retailer for individual 0.9-square-mile area “cells,” aggregated to the school district level and weighted by population. School district average retail marijuana store proximity was categorized as less than 1 mile, 1 mile to 5 miles, and more than 5 miles for analysis.

### Data analysis

We used the Pearson χ^2^ test to determine whether youth demographic characteristics, marijuana use, living with an adult who uses marijuana, or number of and proximity to retail marijuana stores were associated with exposure to marijuana advertising. We used multiple logistic regression models to estimate the adjusted associations between these measures and any marijuana advertising exposure. Models were constructed separately for 8th and 11th graders and included any past-month exposure to marijuana advertising as the dependent variable and the following independent variables: gender, sexual orientation, race/ethnicity, community setting, current marijuana use, living with an adult who uses marijuana, and average proximity to retail marijuana stores in the school district. Number of retail marijuana stores in the school district was not included in models because of high correlation with store proximity (*r* = 0.71).

We conducted analyses with Stata version 13.0 (StataCorp LLC), using procedures that took into account the survey design and weights. The .05 level of significance was used. This study was determined by the Oregon Public Health Division/Multnomah County Health Department Institutional Review Board to be exempt from review per 45 CFR 46.101(b)(4).

## Results

Respondent unweighted demographics are shown in Table 1. The most frequently reported types of advertising exposure (Table 2) were storefronts (37.9% of 8th graders; 43.5% of 11th graders) and online (37.2% for 8th graders; 49.5% for 11th graders). Exposure to billboards was reported by more than one-third of 8th and 11th graders (33.1% and 38.4%, respectively), followed by on the sidewalk (28.6% for 8th graders; 35.0% for 11th graders) and in magazines or newspapers (17.9% for 8th graders; 21.4% for 11th graders). About 1 in 20 8th graders (5.6%) and nearly 1 in 10 11th graders (8.4%) reported past-month exposure to all 5 advertising types assessed.

**Table 1:** Characteristics of Oregon Healthy Teens Survey Respondents, 2017

Characteristic	8th Graders		11th Graders	
	Sample Size^a^	Unweighted %	Sample Size^a^	Unweighted %
**Total**	14,852	100.0	11,895	100.0
**Gender**				
Female	7,037	47.4	5,728	48.2
Male	6,971	46.9	5,430	45.6
Transgender	52	0.4	47	0.4
Nonbinary/gender nonconforming^b^	716	4.8	650	5.5
I do not know what this question is asking	76	0.5	40	0.3
**Sexual orientation**				
Straight	11,389	82.9	9,360	82.6
Lesbian or gay	199	1.5	238	2.1
Bisexual	830	6.0	868	7.7
Something else	551	4.0	449	4.0
Don’t know/not sure	769	5.6	414	3.7
**Race/ethnicity**				
Non-Hispanic White	8,415	59.1	7,437	64.5
Non-Hispanic American Indian or Alaska Native	555	3.9	252	2.2
Non-Hispanic Asian	556	3.9	434	3.8
Non-Hispanic Black or African American	323	2.3	244	2.1
Non-Hispanic Native Hawaiian or other Pacific Islander	136	1.0	104	0.9
Non-Hispanic multiple races^c^	115	0.8	99	0.9
Non-Hispanic other race	491	3.5	274	2.4
Hispanic or Latino ethnicity (any race)	3,651	25.6	2,684	23.3
**Community setting^d^**				
Urban	7,147	55.7	5,762	51.3
Rural	5,176	40.4	5,034	44.8
Frontier	501	3.9	448	4.0
**Current marijuana use**				
Yes	855	6.5	2,224	20.3
No	12,369	93.5	8,706	79.7
**Adult lives in house who uses marijuana**				
Yes	2,290	17.6	2,058	19.3
No	10,712	82.4	8,599	80.7
**Number of retail stores in school district**				
No stores	3,572	24.1	2,728	22.9
1 to 3 stores	4,534	30.5	4,382	36.8
≥4 stores	6,746	45.4	4,785	40.2
**Average proximity to nearest retail marijuana store^e^**				
>5.0 miles	3,395	22.9	2,633	22.1
1.0 to 5.0 miles	9,452	63.6	8,062	67.8
<1.0 mile	2,005	13.5	1,200	10.1

a Sample sizes for some demographic subgroups may not add to the total sample size because of missing data.

b Nonbinary/gender nonconforming category included specific responses: gender nonconforming, genderqueer, gender fluid, intersex/intergender, something else fits better, or I am not sure of my gender identity.

c Non-Hispanic multiple races included respondents who selected more than one race and declined to respond to a question asking them to select a race that best represents them.

d Student zip code and related urban, rural, or frontier designation were missing for 2,028 8th graders and 651 11th graders.

e School district–level, population-weighted average proximity to nearest retail marijuana store in March 2017.

**Table 2:** Past-Month Exposure to Marijuana Advertising Among Youths by Source, Oregon Healthy Teens Survey, 2017

Exposure^a^	8th Graders		11th Graders	
	n	Weighted^b^ % (95% CI)	n	Weighted^b^ % (95% CI)
**Total**	14,852		11,895	
**During the past 30 days, have you seen an advertisement for marijuana products or stores**				
In a magazine or newspaper	2,291	17.9 (16.7–19.2)	2,259	21.4 (20.0–23.0)
On a storefront	4,832	37.9 (35.1–40.8)	4,606	43.5 (41.1–46.0)
Online on your cellphone, tablet, or computer through email, websites, or social media	4,787	37.2 (35.9–38.6)	5,207	49.5 (47.8–51.1)
On a billboard	4,160	33.1 (29.7–36.8)	3,966	38.4 (36.2–40.8)
On the sidewalk (like signs or people wearing or waving signs)	3,662	28.6 (26.4–30.9)	3,655	35.0 (33.0–37.1)
**Number of exposure sources**				
No or unknown exposure	6,780	46.6 (43.8–49.4)	4,382	37.1 (34.7–39.5)
1 source of exposure	2,486	16.4 (15.4–17.5)	1,951	15.8 (14.8–16.9)
2 sources of exposure	2,134	13.9 (13.1–14.7)	1,951	16.1 (14.9–17.3)
3 sources of exposure	1,642	11.0 (10.1–12.1)	1,568	13.2 (12.3–14.2)
4 sources of exposure	998	6.5 (5.6–7.5)	1,079	9.4 (8.5–10.4)
All 5 sources of exposure	812	5.6 (4.8–6.6)	964	8.4 (7.5–9.3)

a Exposure to specific advertising types and number of exposure sources include don’t know/not sure responses in the denominator.

b Weighting included adjustment of participants by grade to school totals, then to county totals, and then to state total enrollment for the 2015–16 school year for schools eligible for participation.

About three-quarters of 8th graders (72.2%) and 11th graders (78.1%) reported seeing any advertising for marijuana products or stores in the past 30 days (Table 3). Among both 8th and 11th graders, exposure to any marijuana advertising in the past month significantly differed by all demographic characteristics assessed in bivariate analysis, including gender, sexual orientation, race/ethnicity, community setting, and current marijuana use. Advertising exposure was also significantly higher among youths who lived with an adult who used marijuana and youths in school districts with high numbers of retail marijuana stores or a closer average proximity to retail marijuana stores.

**Table 3:** Past-Month Exposure to Any Marijuana Advertising^a^, By Demographics and Exposures, Oregon Healthy Teens Survey, 2017

Characteristic	8th Graders				11th Graders			
	n	Weighted % (95% CI)	*P* b	aOR^c^ (95% CI)	n	Weighted % (95% CI)	*P* b	aOR^c^ (95% CI)
**Overall**	11,149	72.2 (69.8–74.4)	NA		9,684	78.1 (76.3–79.7)	NA	
**Demographics**								
**Gender**	11,105	100	NA		9,661	100	NA	
Male	5,170	68.1 (65.3–70.8)	<.001	1 [Reference]	4,383	73.2 (70.8–75.4)	<.001	1 [Reference]
Female	5,370	75.2 (72.7–77.5)		**1.38 (1.23–1.55)**	4,738	82.1 (80.2–83.7)		**1.62 (1.46–1.80)**
Nonbinary/gender nonconforming	565	80.1 (74.4–84.7)		1.31 (0.89–1.95)	540	82.6 (78.0–86.4)		1.26 (0.89–1.78)
**Sexual orientation**	10,984	100	NA		9,598	100	NA	
Straight	9,098	70.5 (68.4–72.6)	<.001	1 [Reference]	7,934	77.1 (75.2–79.0)	<.001	1 [Reference]
Lesbian, gay, or bisexual	1,886	80.4 (76.8–83.5)		**1.56 (1.27–1.93)**	1,664	82.6 (80.3–84.7)		**1.26 (1.02–1.54)**
**Race/ethnicity**	10,770	100	NA		9,429	100	NA	
Non-Hispanic White	6,596	73.5 (70.9–76.0)	.03	1 [Reference]	6,240	80.7 (79.3–82.1)	<.001	1 [Reference]
Non-Hispanic American Indian or Alaska Native	406	71.9 (67.5–76.0)		0.96 (0.70–1.33)	192	75.4 (65.9–82.9)		0.84 (0.50–1.41)
Non-Hispanic Asian	438	72.0 (63.2–79.4)		0.77 (0.55–1.08)	353	71.4 (65.7–76.5)		**0.47 (0.36–0.62)**
Non-Hispanic Black or African American	223	74.3 (65.5–81.5)		0.90 (0.65–1.26)	187	79.6 (71.1–86.1)		0.86 (0.53–1.40)
Non-Hispanic Native Hawaiian or other Pacific Islander	97	79.9 (66.2–89.0)		0.94 (0.47–1.91)	82	77.7 (61.9–88.2)		0.84 (0.36–1.94)
Non-Hispanic multiple or other races^d^	437	73.3 (68.6–77.6)		0.81 (0.64–1.03)	285	77.5 (70.9–82.9)		**0.69 (0.48–0.99)**
Hispanic or Latino ethnicity (any race)	2,573	68.6 (65.9–71.3)		**0.81 (0.71–0.92)**	2,090	71.5 (67.3–75.4)		**0.59 (0.49–0.71)**
**Community setting^e^**	9,902	100	NA		9,247	100	NA	
Urban	5,500	75.9 (72.8–78.8)	<.001	1 [Reference]	4,729	81.1 (78.9–83.1)	<.001	1 [Reference]
Rural	4,070	70.4 (67.6–73.1)		0.92 (0.78–1.09)	4,156	75.6 (72.9–78.1)		0.83 (0.69–1.01)
Frontier	332	53.6 (43.9–63.0)		**0.52 (0.35–0.78)**	362	64.6 (60.6–68.4)		**0.60 (0.44–0.82)**
**Marijuana Use and Exposure in the Home**								
**Current marijuana use (past 30 days)**	11,048	100	NA		9,629	100	NA	
No	10,301	71.2 (68.8–73.4)	<.001	1 [Reference]	7,616	76.5 (74.5–78.4)	<.001	1 [Reference]
Yes	747	84.1 (80.3–87.3)		**1.57 (1.24–1.98)**	2,013	83.5 (80.7–85.9)		**1.53 (1.24–1.90)**
**Lives with adult who uses marijuana**	10,939	100	NA		9,406	100	NA	
No	8,926	69.6 (67.2–71.9)	<.001	1 [Reference]	7,536	76.9 (74.8–78.8)	<.001	1 [Reference]
Yes	2,013	82.8 (80.1–85.2)		**1.85 (1.57–2.19)**	1,870	83.1 (80.5–85.4)		1.21 (0.99–1.48)
**Retail Marijuana Store Exposure**								
**Number of retail stores in school district^f^**								
No stores	2,598	65.8 (61.8–69.6)	<.001	NA	2,200	71.4 (66.7–75.7)	<.001	NA
1 to 3 stores	3,444	69.0 (66.8–71.2)			3,473	78.2 (75.3–80.9)		
≥4 stores	5,107	78.7 (75.1–81.9)			4,011	82.2 (80.0–84.2)		
**Average proximity to nearest retail marijuana store^g^**								
>5.0 miles	2,462	66.3 (62.1–70.3)	<.001	1 [Reference]	2,111	71.9 (67.3–76.0)	<.001	1 [Reference]
1.0 to 5.0 miles	7,178	71.1 (69.5–72.7)		1.16 (0.91–1.48)	6,607	78.4 (76.6–80.1)		1.26 (0.99–1.60)
<1.0 mile	1,509	85.2 (79.0–89.8)		**2.46 (1.43–4.23)**	966	86.7 (83.5–89.3)		**2.56 (1.59–3.21)**

Abbreviation: aOR, adjusted odds ratio; NA, not applicable.

a Any advertising exposure excludes don’t know/not sure responses; boldface aORs indicate significance (*P* < .05).

b *P* value, based on Pearson χ^2^ test, was used to determine whether the proportion exposed to advertising is equal across subgroups for a given characteristic.

c aORs are adjusted for all characteristics listed in the table as indicated, from a model with a main exposure variable of average proximity to nearest retail marijuana store.

d Non-Hispanic multiple races included respondents who selected more than 1 race and declined to respond to a question asking them to select a race that best represents them.

e Community setting was assigned by using student home zip code; 13.7% of 8th graders and 5.5% of 11th graders were not assigned because of missing zip code information.

f aORs in this table are from a logistic regression model with average proximity to nearest retail marijuana store as a primary exposure variable. Similarly aORs from a separate model using number of retail stores as the exposure variable with no stores as reference group were 1.05 (95% CI, 0.86–1.29) for 1 to 3 stores and 1.74 (95% CI, 1.36–2.22) for 4 or more stores among 8th graders, and 1.28 (95% CI, 0.98–1.67) for 1 to 3 stores and 1.59 (CI, 1.24–2.03) for 4 or more stores among 11th graders.

g School district–level, population-weighted average distance to the nearest retail marijuana store location in March 2017.

In logistic regression models for 8th and 11th graders (Table 3), the adjusted odds ratio (aOR) of advertising exposure in the past month were significantly higher (*P* < .05) for girls compared with boys (aOR, 1.38 for 8th graders and 1.62 for 11th graders); for LGB youths compared with straight youths (aOR, 1.56 for 8th graders and 1.26 for 11th graders); for youths who had used marijuana in the past 30 days compared with nonusers (aOR, 1.57 for 8th graders and 1.53 for 11th graders); for youths living with an adult who uses marijuana compared with those without an adult marijuana user in their home (aOR, 1.85 for 8th graders); and for youths in school districts with an average retail marijuana store proximity of less than 1 mile compared with those where the nearest marijuana retailer is more than 5 miles away on average (aOR, 2.46 for 8th graders and 2.56 for 11th graders). Lower odds of ad exposure in the past month were observed for Hispanic or Latino youths compared with non-Hispanic White youths (aOR, 0.81 for 8th graders and 0.59 for 11th graders) and youths living in a frontier community compared with those living in an urban area (aOR, 0.52 for 8th graders and 0.60 for 11th graders).

## Discussion

Three years after Oregonians voted to legalize retail marijuana sales, reported exposure to marijuana advertising appears common among youths: most youths report seeing ads, and a large proportion of those exposed to ads reported seeing them in multiple ways. Exposure reported by Oregon youths in 2017 is higher than national estimates (58.7%) reported by Dai in 2014–2015, which did not specifically report on states with legal marijuana markets (4). Conversely, youth-reported exposure in Oregon was lower than levels reported by Whitehill et al (93.6%) from a 2018 online survey of 469 adolescents aged 15 to 19 years residing in 4 states with legal retail marijuana (not including Oregon); however, exposure to social media sources of advertising in that study was not limited to the past 30 days (9). Regardless of the exact level of exposure, these exposures to marijuana advertising are concerning given the scientific consensus that tobacco promotions cause youth tobacco use initiation (1,10), and alcohol advertising increases the likelihood of alcohol use initiation and heavier drinking if youths already use alcohol (2). Despite not observing significant increases in youth marijuana use following legalization in Oregon (11), marijuana advertising could work in the longer term to similarly increase the likelihood of initiation and heavier use among youths by fostering positive attitudes and expectations of substance use (1).

Marijuana advertising exposure was greater among some groups of youths, potentially putting them at increased risk for initiation. Higher odds of exposure among girls is consistent with a study on e-cigarette advertising that indicated that female youths may be more aware of product advertising in general (12). Youths who identified as LGB were more likely than straight youths to report seeing ads, which may be of particular concern given high rates of current marijuana use within the LGB community: 33% of LGB adults in Oregon currently used marijuana in 2014–2015 compared with 11% of all adults (11). Exposure to advertising was not significantly higher among communities of color compared with non-Hispanic White youths and was significantly lower among youths identifying as Hispanic or Latino. The potential “protective effect” of Hispanic or Latino ethnicity warrants further consideration. However, given historical targeting and relatively higher exposure to advertising from the tobacco and alcohol industries among vulnerable populations (13,14), continued monitoring of advertising exposure among LGB and racial/ethnic minority youths is warranted as the marijuana industry grows from cottage to corporate (15).

Community settings and retail presence may play an important role in advertising exposure. Youths living outside of urban settings were less exposed, which could reflect limited potential for any type of advertising exposure from billboards and storefronts given fewer retail stores generally. In models that controlled for community setting, youths in school districts with closer average proximity to retail marijuana stores were more likely to see ads for marijuana — more than 8 in 10 youths in either grade were exposed within districts where the average proximity to a retail store was less than 1 mile. Communities in Oregon are allowed to ban retail stores, and this may have reduced exposure to advertising for some youths, especially where contiguous geographic areas have done so. Local or state regulation of retail density may play a role in limiting advertising exposure not only by reducing storefront advertising but also by limiting market competition. Washington is the only state to implement a cap on the number of retail licenses allowed in order to support regulation of retail store density (16); the effect of a license cap on community-level store density, associated advertising, and ultimately youth marijuana perceptions and use will require further study. Limiting placement of retailers may limit exposure to their advertising.

Retail marijuana storefronts were among the leading source of advertising seen by youths. While Oregon restricts advertising deemed attractive to minors (ie, features cartoons, images of minors, symbols or celebrities commonly used to market to minors) (17), little else in the state’s rules curbs the influence of retail storefronts on social norms. Washington State, on the other hand, limits stores to 2 signs measuring a maximum of 1,600 square inches that may contain only the trade name, location, and nature of the business (18).

A large proportion of youths in our study reported exposure to online marijuana advertising, including a substantial increase from 8th graders (37.2%) to 11th graders (49.5%) that may reflect increases in media consumption and/or increase in marijuana use with age. This high level of online exposure persists despite state-level regulations that restrict internet advertising to locations where at least 70% of the audience is 21 or older (known as the 70/30 rule) (17), and voluntary policies such as Facebook’s restrictions on advertising illegal, prescription, or recreational drugs (19) and Google’s ban on ads for substances that induce highs (20). Digital marketing through social media — including peer-to-peer transmission through sharing and “likes” — is concerning given the potential for influencing young people and the difficulty of regulating and enforcing restrictions (21,22). Consequently, public health education and countermarketing efforts should include a social media component to reach youths in the digital environment.

About one-third of youths reported exposure to marijuana advertising on billboards and on the sidewalk (eg, signs and people waving or wearing signs). While billboard advertising is restricted by the aforementioned 70/30 rule and state advertising rules provide examples of restricted content, such as images of cartoon characters or toys, this may not be enough to reduce the appeal to youths. A recent letter to the editor in the *Oregonian* newspaper highlights the potential effect of billboards even without youth-oriented images: “Now when I am driving with my 6- and 7-year-old children, I need to explain what ‘Damn $5 a gram’ or ‘celebrate 420’ mean” (Figure 2 is a photograph of a related billboard) (23). In contrast, Colorado allows billboards as fixed signs on a marijuana store’s lot to identify the location but prohibits other forms of outdoor advertising, such as sign spinners and sandwich boards (24).

The American Public Health Association both urges restrictions on marijuana advertising as a public health priority and acknowledges the First Amendment protections of corporate free speech that make marketing regulations difficult (25). A recent analysis of marijuana regulatory frameworks in 4 US states echoes the difficulty of applying tobacco control best practices to advertising restrictions for marijuana, and asserts that existing controls are “limited and ineffective” for preventing youth access and use (26). Those authors further suggest that restrictions on advertising that targets youths may be allowed, and the continued status of marijuana as illegal under federal law may provide states with more regulatory flexibility (26). While outdoor advertising restrictions in Colorado and Washington offer examples of such regulatory flexibility, this latitude is likely state dependent. In Oregon, the limited restrictions on marijuana advertising may be a byproduct of the free speech guarantee in the state constitution, which is considered one of the nation’s strongest and is broader in scope than the First Amendment to the US Constitution (27).

These barriers to advertising regulations and young people’s high exposure to advertising highlight the need for other elements of a comprehensive approach to youth marijuana use prevention, consistent with effective approaches for alcohol and tobacco use prevention. Comprehensive approaches include implementing price controls, developing licensing and zoning laws to restrict and monitor licenses and licensees, restricting public consumption, and designing countermarketing messages (28,29). Our results support the consideration of specific groups of youths (eg, female, LGB) for marijuana countermarketing. Further, complementary parent education campaigns may be warranted given that 8th and 11th graders in our study had higher reported exposure to advertising if someone in the home (eg, parents) used marijuana. For example, the Oregon Health Authority developed the “Talk With Them/Habla Con Ellos” education campaign to support parents and educators in conversations with youths about marijuana. Finally, youth marijuana use should not be addressed in isolation; a truly comprehensive approach should also include tobacco, alcohol, and other drug use prevention given the high prevalence of substance co-use among both youths and young adults in the United States (30,31).

This study had several limitations. First, awareness of marijuana advertising may have been enhanced because this is a new and politically charged topic. Awareness and recollection may diminish over time as retail marijuana and associated advertising become normalized rather than actual changes occurring in the amount of advertising. Second, marijuana advertising questions were added to the youth survey in 2017, so we cannot assess how exposure changed with the opening of the retail market. Given survey space constraints, ad types assessed had to be prioritized and did not include all potential community exposures (eg, those on bus shelters, park benches, transit stations). Third, we used small-area population estimates to describe retail exposure as a population-weighted average by school district; some of Oregon’s school districts span large areas and exposure for individual youths could vary considerably by intradistrict location. Last, we found increased odds of marijuana ad exposure among youths who use marijuana; however, we cannot assert the direction of the association (ie, advertising exposure contributes to marijuana use or marijuana use primes youths to notice advertising). Longitudinal studies are needed to more fully understand this association.

Most youths reported exposure to marijuana advertising in Oregon’s legal retail market, especially youths who are female, LGB, or in school districts with a close average proximity to retail marijuana stores. Different types of advertising may have different potential for regulation. While some sources of youth advertising exposure may be difficult to regulate and enforce (eg, online), others may be within the purview of state authority (eg, billboards, storefronts) depending on state-specific interpretation of free speech protections. States may consider this information both as justification for regulating advertising practices and for selecting specific youth demographic groups to target with prevention messages.
